# Species-Level Salivary Microbial Indicators of Well-Resolved Periodontitis: A Preliminary Investigation

**DOI:** 10.3389/fcimb.2019.00347

**Published:** 2019-10-11

**Authors:** Aneesha Acharya, Tsute Chen, Yuki Chan, Rory M. Watt, Lijian Jin, Nikos Mattheos

**Affiliations:** ^1^Faculty of Dentistry, The University of Hong Kong, Hong Kong, China; ^2^Dr. D. Y. Patil Dental College and Hospital, Dr. D. Y. Patil Vidyapeeth, Pune, India; ^3^Department of Microbiology, The Forsyth Institute, Cambridge, MA, United States; ^4^Department of Oral Medicine, Infection & Immunity, Harvard School of Dental Medicine, Harvard Medical School, Boston, MA, United States

**Keywords:** saliva, salivary microbiota, oral microbiome, periodontal diseases, 16S rRNA gene sequencing

## Abstract

**Objective:** To profile the salivary microbiomes of a Hong Kong Chinese cohort at a species-level resolution and determine species that discriminated clinically resolved periodontitis from periodontally healthy cases.

**Methods:** Salivary microbiomes of 35 Hong Kong Chinese subjects' under routine supportive dental care were analyzed. All subjects had been treated for any dental caries or periodontal disease with all restorative treatment completed at least 1 year ago and had ≤3 residual pockets. They were categorized based on a past diagnosis of chronic periodontitis into “healthy” (H) or “periodontitis” (P) categories. Unstimulated whole saliva was collected, genomic DNA was isolated, and high throughput Illumina MiSeq sequencing of 16S rRNA (V3-V4) gene amplicons was performed. The sequences were assigned taxonomy at the species level by using a BLASTN based algorithm that used a combined reference database of HOMD RefSeqV14.51, HOMD RefSeqExtended V1.1 and GreenGeneGold. Species-level OTUs were subjected to downstream analysis in QIIME and R. For P and H group comparisons, community diversity measures were compared, differentially abundant species were determined using DESeq2, and disease indicator species were determined using multi-level pattern analysis within the R package “indicspecies.”

**Results:** P subjects were significantly older than H subjects (*p* = 0.003) but not significantly different in their BOP scores (*p* = 0.82). No significant differences were noted in alpha diversity measures after adjusting for age, gender, and BOP or in the beta diversity estimates. Four species; *Treponema sp. oral taxon 237, TM7 sp. Oral Taxon A56, Prevotella sp. oral taxon 314, Prevotella sp. oral taxon 304*, and *Capnocytophaga leadbetteri* were significantly more abundant in P than in the H group. Indicator species analysis showed 7 significant indicators species of P group. *Fusobacterium sp oral taxon 370* was the sole positive indicator of P group (positive predictive value = 0.9, *p* = 0.04). Significant indicators of the H category were *Leptotrichia buccalis, Corynebacterium matruchotii, Leptotrichia hofstadii*, and *Streptococcus intermedius*.

**Conclusion:** This exploratory study showed salivary microbial species could discriminate treated, well-maintained chronic periodontitis from healthy controls with similar gingival inflammation levels. The findings suggest that certain salivary microbiome features may identify periodontitis-susceptible individuals despite clinical disease resolution.

## Introduction

Salivary microbiomes show temporal stability in health (Belstrøm et al., 2016). Discernible microbial community patterns at baseline are shown to predict increased risk of inflammatory disease onset in a skin model (van Rensburg et al., 2015). Similarly, whether some microbial “traits” in the oral microbiome are associated with past disease experience or future risk is not established. Subgingival microbiota is most relevant to periodontitis. Distinguishable subgingival microbial clusters have been associated with active chronic periodontitis (Kirst et al., 2014), but not consistently (Koren et al., 2013). Others found subgingival plaque microbiomes did not vary measurably after periodontal therapy (Schwarzberg et al., 2014). Several investigations have shown that salivary microbial profiles can also differentiate both active caries and periodontal disease (Yang et al., 2011; Belstrøm et al., 2015; Chen H. et al., 2015). One plausible reason for these findings is the passage of microbiota from periodontal pockets and carious lesions into saliva. In salivary flora, the proportion of taxa shared with dental plaque is minor but seems sufficient to discriminate disease. As such, the salivary microbiome composition has been found comparable to pooled subgingival plaque in terms of its “representativeness” of the subgingival microbiota (Belstrøm et al., 2017). Others, however, have noted no significant impact of periodontal disease or periodontal therapy on salivary microbiota and attributed this to a low volume of microbial ingress from the subgingival niche (Yamanaka et al., 2012; Takeshita et al., 2016). A previous study from our group found no significant differences in the salivary burden of specific periodontal pathogen abundances between treated, clinically resolved chronic periodontitis and healthy groups (Acharya et al., 2015). However, if no differences existed in the salivary microbiome assemblages between these two groups was unknown. The host-microbiome link is bi-directional, and as inherent features of oral microbiota affect immune programming (Madhwani and McBain, 2016), they may also associate with susceptibility to infection. A stable “core” oral microbial community structure that persists over years has been demonstrated (David et al., 2014) and such ecological stability is linked closely to an individual's salivary composition (Rosier et al., 2018). Plaque biofilm accumulation and its consequent compositional shift to a dysbiotic state is key to the initiation of periodontitis-associated inflammation in susceptible individuals. In resilient individuals, host-immune traits such as salivary nitrate levels and microbial community traits such as bacteriocin levels or anti-inflammatory cytokine stimulation may sufficiently counter disease-related shifts (Rosier et al., 2018). In a carbohydrate challenge experiment, Benítez-Páez et al. (2014) found a caries-resistant individual did not show microbial community shifts similar to caries-susceptible individuals. Arguably, inherent microbial susceptibility “traits” may be reflected in states of clinically resolved periodontal disease. Salivary microbiome differences between well-treated periodontitis and healthy subjects with comparable periodontal health could potentially reflect stable ecological patterns that predict past or future periodontal disease.

Any analysis of microbiome features is highly sensitive to the level of taxonomic resolution achieved. The standard OTU based taxonomy assignment pipelines for 16s rRNA sequence data provide genus-level resolution and have comprised the dominant approach in saliva microbiome studies using 16s rRNA sequencing (Schloss and Westcott, 2011). A novel pipeline reported a taxonomic resolution to the species–level using oral 16S rRNA reads (Al-Hebshi et al., 2015, 2016). This approach was based on the extended human oral microbiome database (HOMD) (Chen et al., 2010). Improved taxonomic feature resolution may enable better discrimination of microbial community fingerprints. In the present study, we profiled the salivary microbiomes of a Hong Kong Chinese cohort using a species-level resolution approach and sought the discriminant species of treated, well-maintained periodontitis from periodontally healthy cases.

## Materials and Methods

### Subject Selection

39 Hong Kong Chinese (HK) adult subjects enrolled in a routine dental maintenance program as a part of a previous multicenter study were (Acharya et al., 2015). All study procedures were conducted at The Prince Philip Dental Hospital, Hong Kong after obtaining Institutional Ethical Committee Approval (HKU/HA: UW 13359) and were in accordance with the Declaration of Helsinki (World Medical Association, 2013). Subjects who had been treated for any caries and periodontal disease, and had completed all active therapies, with the restorative phase completed for at least 1 year, were eligible for recruitment. Inclusion criteria were: age > 30 years and good general health. Exclusion criteria were: presence of >3 residual pockets with PPD > 4 mm, past or current smoking or tobacco chewing, pregnancy, regular use of any medication, having a history of trauma, dental extractions, oral infections, antibiotic, steroidal or csteroidal anti-inflammatory drug consumption within the past 3 months, known systemic diseases (HIV, tuberculosis, hepatitis, known history of any other infectious disease, diabetes mellitus, ischemic heart disease, hypertension, thyroid or other hormonal disorders, autoimmune disease, and cancer) or history of radiation therapy. Subjects were categorized based on their past dental records. A past diagnosis of chronic periodontitis diagnosis at their initial visit according to clinical criteria by Armitage (1999) was categorized as periodontitis (P). Subjects were categorized as healthy (H) if diagnosed as periodontally healthy at the initial visit.

### Saliva Collection, DNA Purification, and Sequencing

Unstimulated whole saliva was collected during the morning as described before (Acharya et al., 2015). In brief, unstimulated saliva was collected before 11 a.m. in the morning. Subjects had refrained from eating or drinking for a minimum of 1 h before saliva collection. Saliva was allowed to passively pool intraorally and was expectorated into sterile collection tubes, which were kept on ice and immediately transported for storage at −80°C until further analysis. Saliva samples were thawed, equilibrated to room temperature before DNA extraction. Salivary cells were pelleted by centrifugation at 14,000 rpm for 10 min and the supernatant was carefully removed. Salivary genomic DNA was extracted using the column extraction method with the QIAamp DNA Blood Mini Kit®, following the manufacturer's instructions. The DNA concentration was assessed using a NanoDrop® spectrophotometer and a Qubit® Fluorometer, while its integrity was determined using agarose gel electrophoresis. 16S rRNA V3-4 gene amplification and MiSeq (300 base-paired end) sequencing was performed using the standard Illumina dual-indexing protocol (Fadrosh et al., 2014). The primer sequences used were; Forward: CCTACGGGNGGCWGCAG Reverse: TACNVGGGTATCTAATCC.

### Data Analysis

**Figure 1:**
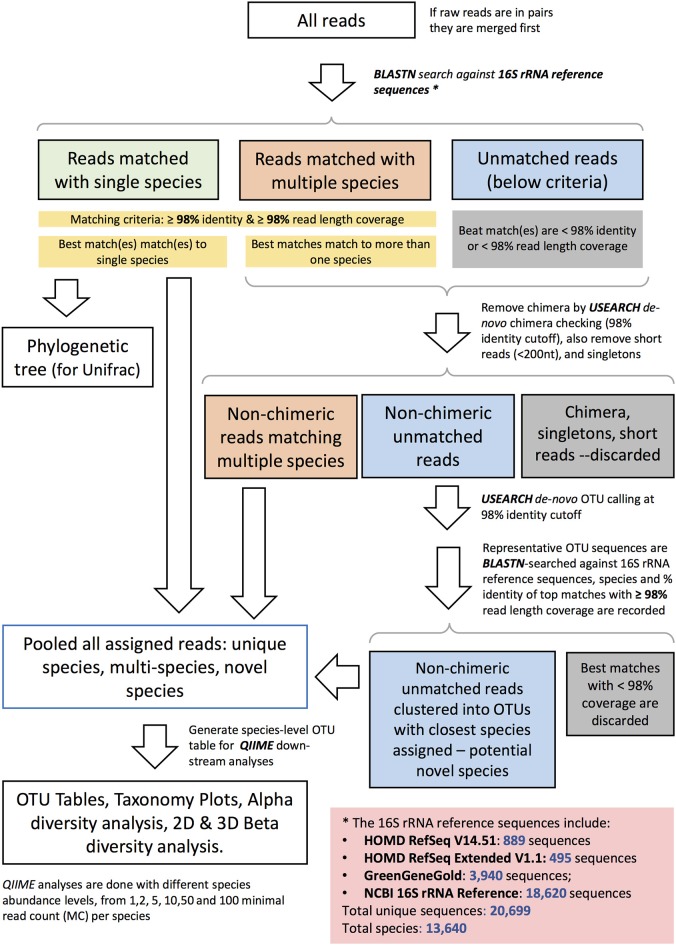
Schematic diagram representing the species–level open reference taxonomy assignment for 16S rRNA Reads.

Sequencing data were quality filtered. Demultiplexing, barcode, linker-primer sequences trimming, and raw reads quality filtering (minimum *Q*-value = 20, with removal of reads with 20% or higher of read original length with low quality, removal of reads contaminated by adapter, with ambiguous or N basecall, and those with low complexity as indicated by 10 consecutive same bases) was done using MISEQ reporter (v 2.0) software. Raw FASTQ sequences were deposited in the SRA BioProject database (BioProject ID Number PRJNA337949). Samples with <500 read counts were excluded in all analysis. Read pairs were merged to single sequences with a script (join_paired_ends.py) provided by the Quantitative Insights into Microbial Ecology (QIIME) package version 1.9.1 (Caporaso et al., 2010), using default settings. The merged reads were then taxonomically assigned to the species-level based on a previously published algorithm (Al-Hebshi et al., 2015), with additional steps to further identify potential novel species. The taxonomy assignment pipeline is depicted in Figure 1. Briefly, merged sequence reads were BLASTN-searched against a panel of full-length 16S rRNA sequences. These consisted of 889 sequences from HOMD RefSeqV14.51, 495 from HOMD RefSeqExtended V1.1, 3,940 from GreenGeneGold, and 18,620 from NCBI 16S rRNA Reference. The version of the 16S rRNA gene reference sequence database used by this manuscript is dated 20170422 and is available online together with the documentation: http://www.homd.org/ftp/NGS_Pipeline/Species_Level_BLASTN/. This combined reference set has a total 20,699 sequences and represents a total of 13,640 oral and non-oral microbial species. The first step was a BLASTN-search with a matching criteria of ≥98% identity and ≥98% read length coverage. Reads matching multiple species or unmatched reads were further processed where *de novo* Chimera were filtered using USEARCH (Edgar, 2010) with (98% identity cutoff), short reads (<200 nt), and singletons were removed. *De-novo* OTUs (operational taxonomy units) were clustered at a 98% identity cutoff. USEARCH (version v8.1.1861_i86linux32) was used for taxonomy assignment and those with <98% match were discarded. If sequences with the same scientific name had different upper-level taxonomy nomenclature in the combined reference set, we choose a single taxonomy with the following priority: HOMD+HOME-EXT, GreenGene Gold, and then NCBI Taxonomy. Manual curation was also performed to resolve issues including mis-spelling, non-alpha numeric characters, and missing information. The algorithm for taxonomy assignment is illustrated in Figure 1 and documented in the online resource: http://www.homd.org/ftp/NGS_Pipeline/Species_Level_BLASTN/20170422/Species_Level_BLASTN_QIIME_Pipeline_Doc_20170422.pdf.

After the taxonomy assignment, species-level OTUs were subject to down-stream bioinformatics analyses, including alpha and beta diversity analyses using the QIIME pipeline. Species-level taxonomic plots were generated. Alpha diversity measures were computed, rarefaction curves were drawn and comparisons made between P and H groups with linear regression controlling for Age, Gender, and BOP as co-predictors. Beta diversity measures, phylogenetic (weighted and unweighted unifrac) and Bray-Curtis metrics were determined, Principal coordinate analysis plots were drawn and comparisons between P and H categories made using permutational multivariate analysis of variance using “Adonis” (R package “vegan” version 2.2.1) (*p*-values generated at 5,000 permutations). Similar comparisons were made to analyse community differences related to Gender, Age group, and BOP.

To determine species associated with subject characteristics, 2 approaches were adopted using the species-level OTU abundance table as input. Firstly, the differential abundance (DA) of species between sample groupings (Periodontal status; P vs. H, Gender: Male vs. Female, Age: >50 years vs. ≤ 50 years, BOP: >25% vs. ≤ 25%) were each determined using DESeq2 with *p*-value adjusted for multiple testing using the Benjamini–Hochberg method (cut-off = adjusted *p* < 0.05) (Love et al., 2014). Secondly, “indicator species” were determined by multi-level pattern analysis. For this purpose, the “multipatt” and “indval.g” functions in the R package “Indicspecies” were used (De Caceres and Legendre, 2009). The indicator approach measures the predictive ability of community features for a group category (McGeoch and Chown, 1998). The indicator statistic represents the probability of group fidelity. Ideal indicator species are characterized by high exclusivity and fidelity. The function “indVal.g” accounts for unbalanced between- group size variances. Indicator species with significant *p*-values (<0.05, computed using 5,000 permutations) were determined (De Cáceres et al., 2012).

## Results

### Clinical Data, Sequencing Output, Processing and Taxonomy Assignment

**Figure 2:**
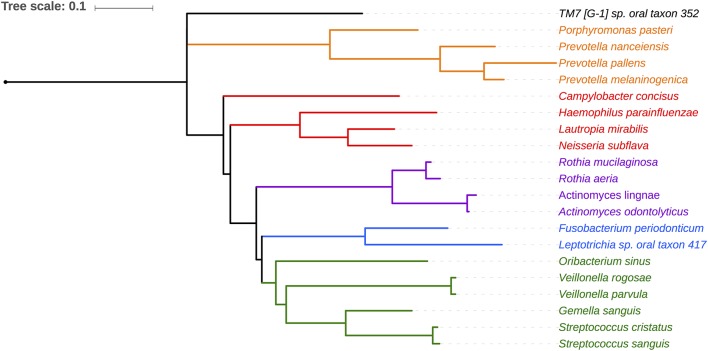
Phylogenetic relationship of 21 species shared by 80% of the subjects. Phylotypes assigned to single HOMD entity are shown. Five phylotypes assigned to multiple species are not shown (*Granulicatella multispecies spp49 2, Streptococcus, multispecies spp1 2, Streptococcus multispecies spp24 14, Streptococcus multispecies spp32 2*).

Salivary microbiomes of 35 subjects were analyzed after 4 subjects were excluded due to having <500 reads per sample. The read count details are presented in Table S1. The clinical and demographic data for these 35 subjects are summarized in Table 1. A total of 63,834 assigned reads and 418 species-level taxa were determined at ≥98% sequence identity and ≥98% read length coverage to the reference sequences. Of these, 56,399 reads were assigned to 358 single species, 7,413 reads were assigned to 51 taxa that contain multiple species due to tied sequence identity to reference sequences of multiple sequences, and 22 were assigned to 6 potential novel species due to <98% sequence identity to all the reference sequences (Table S2). The genera with the most number of taxa assigned with multiple species were *Fusobacterium* (11 species, ranging from 2 to 5 species), *Streptococcus* (11 species, ranging from 2 to 15 species), and *Prevotella* (6 species, ranging from 2 to 4 species). Six novel species were assigned to a very small number (22) of reads: *Flavobacriia* genera, *Bergeyella sp. Oral taxon 907* (97.42% identity) and *Capnocytophaga; multispecies* (96.35% identity), *Clostridiales* multispecies (96.21% identity), *Sporomusaceae multispecies* (87.50% identity), *Leptotrichia multispecies* (97.99% identity), *Neisseria sp. Oral taxon 018* (97.67% identity). The number of species observed per sample ranged from 43 to 186 (mean = 89.1, sd = 29.0). Species shared among individuals were considered as representing a “core microbiome.” Seventy-nine species were shared by 50% individuals and 26 by 80% individuals including species from *Streptococcus, Gemella, Rothia, Actinomyces, Fusobacterium, Leptotrichia, Prevotella, Porphyromonas, Neisseria, Capnocytoph*aga, *Haemophilus and Lautropia* (Figure 2). Five species were present in 95% individuals; *Rothia mucilaginosa, Prevotella melaninogenica, Streptococcus multispecies spp24_14, Veillonella parvula, Haemophilus parainfluenzae* (Table S3).

**Table 1:** Clinical and demographic characteristics.

**Group**	**H** ** (*n* = 20)**	**P** ** (*n* = 15)**	**Comparison** ** P vs. H**	**Total** ** (*n* = 35)**
Mean Age (st.dev) (years)	47.3 (14.5)	60.9 (7.0)	*W* = 62.5, *p* = 0.003^*^	53.8 (13.6)
Gender	15F, 5M	11F, 4M	*X*^2^ = 0.013, *p* = 1	26F, 9M
Mean % BOP positive sites (st.dev)	15.4 (13.8)	16.7 (20.1)	*W* = 157.5, *p* = 0.82	16.0 (15.5)

W, Mann Whitney Wilcoxson's test statistic; X^2^, Chi-squared test statistic;

* *p < 0.05, significant*.

### Community Differences Between Periodontitis and Health Groups

**Figure 3:**
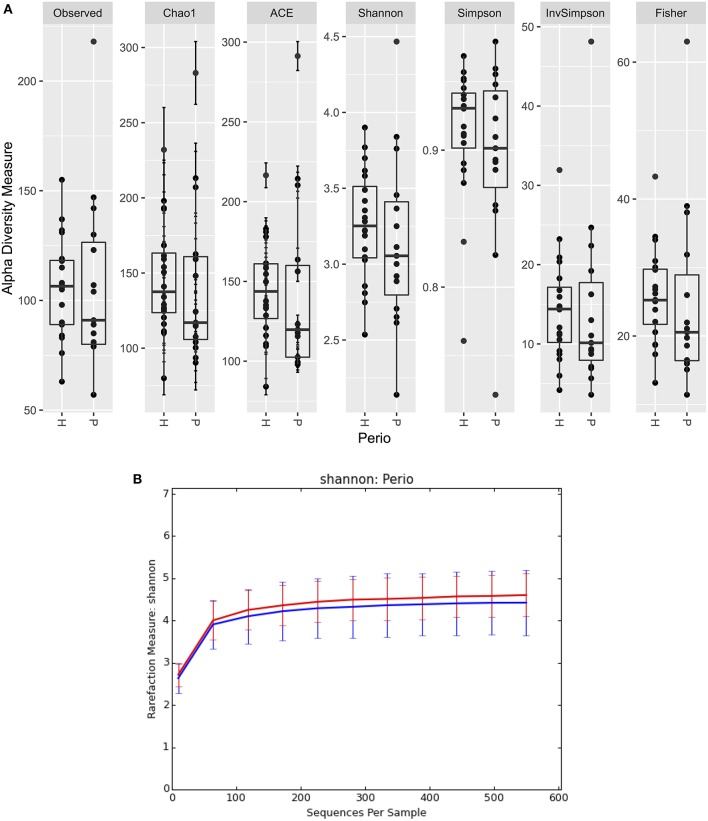
**(A)** Boxplots of alpha diversity measures in P and H groups. **(B)** Rarefaction plot of Shannon alpha diversity in P and H groups. P, blue color; H, red color.

**Figure 4:**
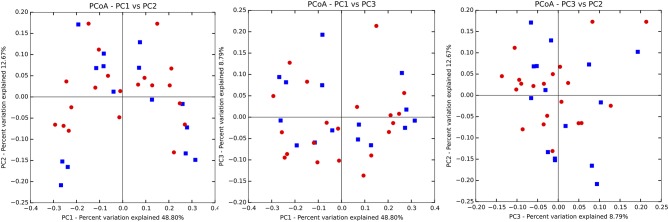
Principal coordinate ordination plots based on the weighted Unifrac beta diversity distance. P, blue color; H, red color. No clustering of P and H samples was evident.

There were no significant differences observed in mean alpha diversity measures for the P and H groups, which was reflected in the rarefaction plots (Figures 3A,B). However, the P group showed lower alpha diversity measures compared to the H group. Linear regression analysis (controlling for the effects of age, gender, and BOP) showed that a periodontal disease status did not have a significant impact on salivary alpha diversity measures (Table 2, Table S4). Comparison of beta diversity measures; Bray–Curtis (*f* = 0.56, *p* = 0.93) and Unifrac measures (unweighted Unifrac: *f* = 0.78, *p* = 0.66; weighted Unifrac: *f* = 0.25, *p* = 0.97) revealed no significant differences in community composition between the P and H groups. Correspondingly, no evident clustering of P or H cases was noted in the ordination plots (Figure 4). Similarly, no significant differences in beta diversity were associated with gender, BOP or Age categories (age > 50 years, age ≤ 50 years).

**Table 2:** Adjusted linear regression coefficients for alpha diversity indices between P vs. H group.

**Diversity metric**	**Coefficient^a^**	**95% CI^a^**	***P*-value^**a**^**	**Variance explained** **(Model R squared)**
Observed species	1.1	−23.0 to 25.7	0.93	0.03
Chao 1	−2.7	−39.4 to 34.0	0.88	0.03
ACE	−8.7	−42.7 to 25.4	0.61	0.04
Shannon	−0.13	−0.5 to 0.3	0.53	0.04
Simpson	−0.01	−0.1 to 0.0	0.73	0.03
Inverse Simpson	−2.3	−8.6 to 4.1	0.47	0.05
Fisher's alpha	−1.5	−9.1 to 6.1	0.68	0.05

*Simple linear regression with predictors Age, BOP, Gender (reference level = F), and Periodontal Status (reference level = H)*.

a *Values for predictor “Periodontal Status”*.

**Figure 5:**
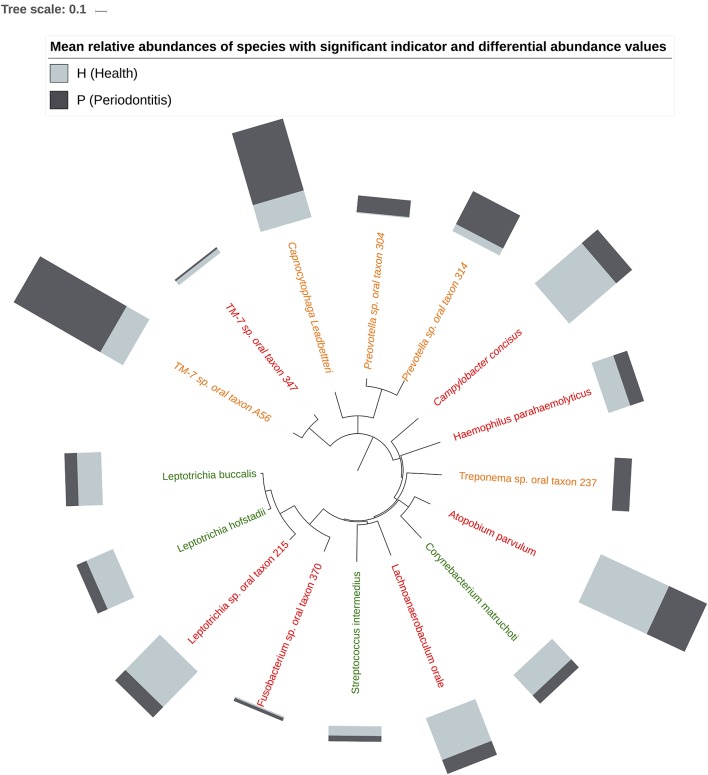
Phylogenetic relationship of Indicator (H indicators in green, P indicators in red) and DA species (in orange). Bars represent mean relative abundances.

Figure 5 shows the mean relative abundances and phylogenetic relationships of the indicator and DA species that discriminated the P and H groups. Using DeSeq2, 4 species were found to be significantly more abundant in the P group as compared to the H group (Table 3, Table S5). These were *Treponema sp. oral taxon 237, TM7 sp. Oral Taxon A56, Prevotella sp. oral taxon 314, Prevotella sp. oral taxon 304, Capnocytophaga leadbetteri*. However, no species had significant differences in abundance between males and females, or between the two age categories. A BOP value of >25% was associated with a significantly higher abundance of *Fusobacterium multispecies spp48_2* (log2 fold change = 2.32, adjusted *p* < 0.001). Indicator analysis showed 7 species as significant indicators of the P group (Table 4, Table S6). Among these, 6 were negative indicators whereas *Fusobacterium sp oral taxon 370* was the only positive indicator. Four species emerged as significant H group indicators (Table 4). *TM7 G.1 sp oral taxon 347* and *Fusobacterium sp oral taxon 370* had low sensitivity but high positive predictive values (>0.9) for P group.

**Table 3:** Differential abundance of significantly different species between P and H groups (DeSEQ2 test).

**Species**	**Log2 fold change** **P vs. H (se)**	**Test statistic**	**Adjusted *p*-value**	**Total reads assigned to species**
*Treponema sp. oral taxon 237*	1.68 (0.41)	4.15	<0.01^*^	56
*TM7 sp. Oral Taxon A56*	1.80 (0.45)	4.01	<0.01^*^	519
*Prevotella sp. oral taxon 314*	1.54 (0.39)	3.94	<0.01^*^	143
*Prevotella sp. oral taxon 304*	1.57 (0.41)	3.86	<0.01^*^	62
*Capnocytophaga leadbetteri*	1.45 (0.42)	3.43	0.02 ^*^	357

* *p < 0.05, significant*.

**Table 4:** Indicator species of P and H groups.

	**Test-statistic**	***p*-value**	**Positive predictive value**	**Sensitivity**	**Total reads assigned to species**
**Group H (number of indicator species** **=** **4)**					
*Leptotrichia buccalis*	0.80	0.01^*^	0.85	0.75	107
*Corynebacterium matruchotii*	0.79	0.03^*^	0.82	0.75	128
*Leptotrichia hofstadii*	0.72	0.04^*^	0.87	0.60	126
*Streptococcus intermedius*	0.71	0.03 ^*^	0.77	0.65	43
**Group P (number of indicator species** **=** **7)**					
*Campylobacter concisus*	0.86	0.02^*^	0.74	1.00	291
*Atopobium parvulum*	0.79	0.03^*^	0.71	0.87	416
*Leptotrichia sp oral taxon 215*	0.76	0.04^*^	0.79	0.73	213
*Lachnoanaerobaculum orale*	0.76	0.04^*^	0.86	0.67	193
*Haemophilus parahaemolyticus*	0.74	0.03^*^	0.82	0.67	127
*TM7 G.1 sp oral taxon 347*	0.56	0.04^*^	0.94	0.33	26
*Fusobacterium sp oral taxon 370*	0.55	0.04^*^	0.90	0.33	16

* *p < 0.05, significant*.

## Discussion

Here, we conducted an exploratory investigation to assess the salivary microbiome in P and H subjects within a Hong Kong Chinese cohort. The pattern of shared “core” species was similar to past reports (Takeshita et al., 2016; Mason et al., 2018) and particularly resonated with a previous report in Chinese subjects (Huang et al., 2011). The use of a smaller, curated reference sequence set may have enabled better accuracy of species-level taxonomical assignment. Arguably, higher taxonomic resolution may improve the discrimination of host trait-microbiota associations. We noted significant differences in the relative abundances of select salivary species and identified several indicator species discriminatory of P and H groups.

The ordination or beta diversity analysis of our data appeared to be in agreement with Takeshita et al. (2016) who found no association between salivary microbiome composition and periodontal disease. However, unconstrained beta diversity ordinations reflect only the major sources of variation in composition. Salivary community diversity measures were not significantly linked to any of the clinical and demographic variables, although a trend for lower alpha diversity was notable in the P group. The small sample and restrictive inclusion criteria very likely limit the detection of differences in microbiome composition related to subject background. As such, most subject characteristics have small effect-sizes on microbiome composition and very few factors like geographical location and lifestyle are known to have large effect sizes (Lazarevic et al., 2010).

P group subjects were significantly older but did not differ in levels of BOP from H subjects and had 3 or less periodontal pockets, reflecting well-resolved periodontitis with low levels of residual disease activity. Treated periodontitis cases can vary in the level of residual periodontal disease. As influx from inflamed subgingival niches is likely to be a major source of periodontitis-associated bacteria in saliva (Saygun et al., 2011; Belstrøm et al., 2015), the selection criteria were designed to exclude P subjects with significant numbers of residual periodontal pockets. Similar levels of residual inflammation and lack of residual pockets in the P group could be one factor why no major ecological differences from H subjects were noted. The most dominant factor in microbiome composition is interpersonal variation (Lazarevic et al., 2010). A large prospective study analyzed microbial communities in periodontal disease and health (Chen et al., 2018) and found disease-linked microbiomes are more heterogeneous in both pre- and post-treatment situations. Further studies are needed to understand the extent to which salivary microbiomes may be stable and resistant to periodontal treatment.

We used two approaches to analyse microbial features that distinguished the P and H categories. The DESeq2 approach utilized here is more robust in small sample sizes as compared to non-parametric approaches. The “indicator species” analysis is based on the “Indicator Value” index (De Caceres and Legendre, 2009) which reflects the association of a feature with grouping category. None of the significant indicator species were “ideal” or had sensitivity and specificity values close to 1. While these would need validation in larger cohorts, this finding may also reflect the difficulty of individual microbial features which strongly reflect a periodontitis phenotype, considering the complex, polymicrobial nature of the disease and variations in both health and disease linked microbiome compositions. Despite comparable levels of gingival health, the P group was distinguishable by specific salivary microbial species, which largely aligned with past reports (Abusleme et al., 2013; Pérez-Chaparro et al., 2014). Among the significantly raised species in P, *Treponema sp. oral taxon 237* has been implicated as a periodontal pathogen (Abusleme et al., 2013). Two *Preovtella* species *Prevotella sp. oral taxon 314* and *Prevotella sp. oral taxon 304* were significantly more abundant in P subjects. *Prevotella* species have previously been associated with both periodontal health and disease. A considerable amount of species and strain level genomic diversity (Ibrahim et al., 2017), as well as variability in their response to periodontal therapy, is documented (Schwarzberg et al., 2014). An increase in salivary anaerobic species in treated periodontitis may imply greater potential systemic risk due to extraoral translocation (Endo et al., 2015; Vieira Colombo et al., 2016). Some *Prevotella* species, in particular, have been linked to new-onset rheumatoid arthritis (Scher et al., 2012). A past study, reported the anaerobic bacillus *Capnocytophaga leadbetteri* in periodontitis and hematological infections but not in periodontally healthy mouths (Ehrmann et al., 2013).

*Fusobacterium sp oral taxon 370* has been linked to fermentative function, and has been found to be raised in periodontal disease (Dabdoub et al., 2016). Furthermore, *Fusobacterium multispecies spp48_2* species was significantly more abundant in subjects with high BOP, independent of past periodontal status. With the exception of *Fusobacterium sp oral taxon 370*, all other P state indicator species were “negative” indicators characterized by greater mean abundance in H subjects. Indicator analysis identified several species that were lower in P as having significant indicator value. Notably, several of these negative indicator species were from genera previously identified as associated with periodontitis (Pérez-Chaparro et al., 2014), which seems somewhat paradoxical considering they were found in lower relative abundance in resolved disease compared to health. Hypothetically, it may reflect differences in microbiome assembly in plaque and saliva caused by periodontal therapy (Chen et al., 2018). *Campylobacter concisus* emerged as a negative indicator for P cases. This species is recognized as oral commensal and implicated in gastric and inflammatory bowel disease (Liu et al., 2018). The oral cavity is considered a potential reservoir (Zhang et al., 2010). It can act as an opportunistic pathogen in immune-inflammatory and epithelial cell dysregulation (Deshpande et al., 2016; Brunner et al., 2018). A rise in its abundance after successful periodontitis therapy has been reported (Tanner et al., 1987), again supporting the premise that periodontal treatment may result in specific differences in microbiome composition. The indicator species *Lachnoanaerobaculum orale* lies within *Lachnospiraceae* which were previously reported by Chen L. et al. (2015) as inversely linked to caries in Chinese subjects. Some *Lachnospiraceae* members are newly recognized in periodontitis and some species correlate with gingival inflammation (Abusleme et al., 2013; Kistler et al., 2013). *Lachnospiraceae* contains many genera that are butyrate producers, a trait associated with anti-inflammatory properties in the gut (Lopetuso et al., 2013) but also with periodontitis progression (Tsuda et al., 2010). The low-abundance species *Atopobium parvulum* has been linked to halitosis (Kazor et al., 2003). Several *Haemophulis* species are members of core oral microbiome (Mok et al., 2017). *TM7 G.1 sp oral taxon 347* was increased in subgingival plaque of active periodontal disease (Griffen et al., 2011). Overall, the indicator analysis seemed in particular agreement with a past report that found subgingival *Capnocytophaga, Corynebacterium, Haemophilus, Leptotrichia*, and *Streptococcus* had significant negative associations with clinical periodontal measures (Camelo-Castillo et al., 2015).

The present findings must be considered in light of the major limitations of the study; the subject groups were small, not balanced for size, demographics and past periodontal disease parameters including disease severity, which limit the generalizability of these results. Importantly, the small sample size of 35 allows only preliminary findings and these require validation in large sampled studies. Because of the primarily exploratory focus of this study, the downstream analysis was based on a minimum read count per species of 1 and aimed to capture the maximal ecological variation feasible. On the same lines, the 6 novel species reported were based on a very small number of reads. These findings may be considered as a basis for a hypothesis that variations in the oral microbiome related to a periodontitis phenotype are retained even after clinical disease resolution. Another question that arises is whether oral species can discriminate periodontally susceptible individuals before clinical disease onset or during very early disease. Longitudinal and interventional investigations in larger cohorts are essential to test such hypotheses. Translational implications of such findings could be the development of improved microbiome-based risk-markers or therapeutics, considering saliva is an attractive sampling biofluid. Other implications include an improved understanding of host-microbiome interactions in the context of periodontal disease. We did not analyse how the salivary ecological composition represented that of the subgingival niche, which should be addressed in future studies. The issues of sampling variance and compositional nature of microbiome data need to be considered when viewing the generalisability of the particular signature taxa, which were based on a single replicate in our study. Technical differences in saliva collection methods, storage, handling, DNA extraction, choice of sequencing technology or 16s rRNA gene hypervariable region, sequencing depth, and bioinformatic pipeline can each cause induce variation in the outcomes (Mascitti et al., 2019) and precludes a generalization of these specific findings. Here, we collected unstimulated saliva, which varies in its microbial profile from stimulated saliva and is shown to harbor a greater diversity of species (Gomar-Vercher et al., 2018). Besides, other influencing factors such as diet, body-mass index, and multiple lifestyle factors which impact microbiome assemblages were not considered in this preliminary analysis and could be a source of confounding. In addition, a single time point sampling precludes the differentiating between stable and dynamic salivary species. The exact biological relevance of the discriminant microbial features also remains unclear. While taxonomical information is valuable, functional aspects of the host-microbiome interaction are central to disease processes. Whole-genome, transcriptome, and metabolome approaches may better answer how saliva microbiome is linked to periodontal susceptibility.

## Conclusion

Species-level analysis of the salivary microbiome in well-resolved periodontitis cases revealed no differences in global community diversity metrics as compared with periodontally healthy subjects. However, differentially abundant and indicator species were discernible between these groups. These findings suggested differences in oral microbiota may persist despite acceptable clinical resolution of periodontitis. The nature and implication of these differences warrant further investigations.

## Ethics Statement

This study was carried out in accordance with the recommendations of Declaration of Helsinki, World Medical Association with written informed consent from all subjects. All subjects gave written informed consent in accordance with the Declaration of Helsinki. The protocol was approved by the Institutional Review Board of The University of Hong Kong/Hospital Authority Hong Kong West Cluster (HKU/HA HKW IRB) (ref: IRB number UW 13359).

## Author Contributions

AA aided the study design, collected the samples and clinical data, performed the laboratory steps, statistical analysis, and compiled the study findings as a manuscript. TC performed the sequence data processing, taxonomy assignments to the species-level and edited the manuscript. YC contributed to the study design and strategy for the statistical analysis. RW contributed to the study concept and design, supervision of laboratory steps, manuscript preparation and revision. NM contributed to the study concept and design, overall supervision, clinical management, and revised the manuscript. LJ contributed to the study concept and manuscript editing.

### Conflict of Interest

The authors declare that the research was conducted in the absence of any commercial or financial relationships that could be construed as a potential conflict of interest.
